# Normative Database of the Superior–Inferior Thickness Asymmetry for All Inner and Outer Macular Layers of Adults for the Posterior Pole Algorithm of the Spectralis SD-OCT

**DOI:** 10.3390/jcm12247609

**Published:** 2023-12-11

**Authors:** Ana Palazon-Cabanes, Begoña Palazon-Cabanes, Jose Javier Garcia-Medina, Aurora Alvarez-Sarrion, Monica del-Rio-Vellosillo

**Affiliations:** 1 Department of Ophthalmology, Hospital Virgen del Castillo, 30510 Murcia, Spain; a.palazoncabanes@gmail.com; 2 Department of Neurology, Hospital de la Vega Lorenzo Guirao, 30530 Murcia, Spain; abega_ct@hotmail.com; 3 Department of Ophthalmology, General University Hospital Morales Meseguer, 30008 Murcia, Spain; 4 Department of Ophthalmology and Optometry, University of Murcia, 30120 Murcia, Spain; aurora.alvarezopt@gmail.com; 5 Ophthalmic Research Unit “Santiago Grisolia”, 46010 Valencia, Spain; 6 Spanish Net of Inflammatory Diseases RICORS, Institute of Health Carlos III, 28029 Madrid, Spain; 7 Department of Anesthesiology, University Hospital Virgen de la Arrixaca, 30120 Murcia, Spain; monica.delrio@um.es; 8 Department of Surgery, Obstetrics and Gynecology and Pediatrics, University of Murcia, 30120 Murcia, Spain

**Keywords:** normative database, asymmetry, hemispheres, thickness, macula, layer, posterior pole algorithm, optical coherence tomography, glaucoma, neuro-ophthalmology

## Abstract

Background: This study aims to establish a reference for the superior–inferior hemisphere asymmetry in thickness values for all macular layers for the posterior pole algorithm (PPA) available for the Spectralis SD-OCT device. Methods: We examined 300 eyes of 300 healthy Caucasian volunteers aged 18–84 years using the PPA, composed of a grid of 64 (8 × 8) cells, to analyze the thickness asymmetries of the following automatically segmented macular layers: retinal nerve fiber layer (RNFL); ganglion cell layer (GCL); inner plexiform layer (IPL); inner nuclear layer (INL); outer plexiform layer (OPL); outer nuclear layer (ONL); retinal pigment epithelium (RPE); inner retina; outer retina; complete retina. Mean ± standard deviation and the 2.5th and 97.5th percentiles of the thickness asymmetry values were obtained for all the corresponding cells. Results: All the macular layers had significant superior–inferior thickness asymmetries. GCL, IPL, INL, ONL and RPE showed significantly greater thicknesses in the superior than the inferior hemisphere, whereas RNFL and OPL were thicker in the inferior hemisphere. The largest differences between hemispheres were for RNFL and ONL. Conclusions: This is the first normative database of macular thickness asymmetries for the PPA and should be considered to distinguish normal from pathological values when interpreting superior–inferior macular asymmetries.

## 1. Introduction

Asymmetry in the macular structure due to changes in different macular layer thicknesses has been demonstrated in several diseases [1,2,3,4,5,6], especially in glaucoma [7,8,9,10,11]. Glaucoma is a leading cause of irreversible blindness worldwide and its incidence is increasing over time [12]. Glaucoma is a multifactorial disease characterized by the progressive apoptosis of retinal ganglion cells and the degeneration of their axons. This leads to the asymmetric thinning of the innermost macular layers, accompanied by corresponding visual field defects that initially tend to respect the horizontal midline [7,8]. It has also been suggested that the thickness of the inner nuclear layers and the different outer macular layers can also change in glaucoma, although the findings of previous studies are still controversial [8,9,10,11]. As this condition remains asymptomatic until advanced stages, regular screening examinations are essential, mainly in high-risk populations. However, the sensitivity and specificity of the glaucoma screening tests available today are low [13,14]. Therefore, the early detection of thickness asymmetry of the different macular layers between the superior and inferior hemispheres could constitute a novel strategy to improve the early diagnosis and treatment of glaucoma [15].

The Spectralis spectral domain optical coherence tomography (SD-OCT) device has several exploration protocols for the macula. One of them, the posterior pole algorithm (PPA), was specifically designed to follow-up patients with suspected or diagnosed glaucoma [16]. This protocol allows the automatic segmentation of the different macular layers and is able to detect retinal thickness differences of up to 30 microns between the corresponding points of the superior and inferior hemispheres of the macula [16]. However, previous studies have reported that thickness asymmetry is also present in healthy subjects [7,17]. Hence, a simple thickness asymmetry analysis is not specific enough to distinguish the physiological asymmetries from those that can be early signs of glaucoma.

The aim of the present study was to determine the normal range of asymmetries of the macular thicknesses between the corresponding points of the superior and inferior hemispheres in a healthy Caucasian population using the PPA of the Spectralis SD-OCT. To the best of our knowledge, such a normative database has not yet been published.

## 2. Materials and Methods

This is an observational cross-sectional study that included 300 eyes of 300 healthy Caucasian adults. Only one eye per patient was randomly selected. Volunteers were recruited in the Department of Ophthalmology at the University General Hospital Reina Sofia in Murcia, Spain. They were chosen proportionally according to gender and age to obtain a representative sample population, and included 50 people (25 males, 25 females) in all six established age groups (18–29, 30–39, 40–49, 50–59, 60–69 and 70–85 years). The inclusion criteria were as follows: aged 18–85 years, Caucasian ethnicity, normal ophthalmological examination and normal peripapillary retinal nerve fiber layer (pRNFL) thickness. The exclusion criteria were as follows: adjusted intraocular pressure (IOP) > 21 mmHg, cup-to-disc ratio > 0.4, sphere ≥ 5 diopters and/or cylinder ≥ 2 diopters, any ocular surgery within the last 6 months, any previous or current ocular disease (glaucoma, diabetic retinopathy, uveitis, amblyopia, etc.), any neuropsychiatric diseases or any media opacities leading to a signal strength of the OCT images below 25.

The study protocol adhered to the ethical principles of the Declaration of Helsinki and was approved by the Local Ethics Committee at the University General Hospital Reina Sofia in Murcia, Spain (protocol number 03/19). Subjects were informed about the study and informed consent was obtained from them all before enrollment.

A comprehensive ophthalmologic examination was carried out on all the participants, including autorefractometry (NIDEK ARK-710A; NIDEK, Aichi, Japan), visual acuity, IOP determination pneumatic tonometry, slit-lamp biomicroscopy and fundus examination, as well as measurement of axial length, keratometry (IOL Master; Carl Zeiss Meditec Inc., Dublin, CA, USA) and pachymetry (Specular Microscope EM-3000; Tomey; Phoenix, AZ, USA). The PPA and the optic disk circle protocols of the Spectralis (Heidelberg Engineering, Heidelberg, Germany; software version 6.0) were employed to acquire OCT images of the macula and optic disk, respectively. All the OCT examinations were performed by the same experienced ophthalmologist (A.P.C).

The PPA scans a macular cube measuring 30° × 25°, centered on the fovea and oriented using fovea–disk alignment [16]. The results are shown on a macular grid that is divided into 64 cells, each measuring 3° × 3°, which are distributed in eight rows and eight columns (8 × 8 PPA). Cell numbering is established from inferior to superior and from temporal to nasal. Thus, the nomenclature is specular between eyes. Cell 1.1 is the most inferior—temporal cell and cell 8.8 is the most superior–nasal, both in right and left eyes. Using the automatic segmentation tool of this protocol, for each cell, we obtained the thickness values of the following macular layers: retinal nerve fiber layer (RNFL); ganglion cell layer (GCL); inner plexiform layer (IPL); inner nuclear layer (INL); outer plexiform layer (OPL); outer nuclear layer (ONL); retinal pigment epithelium (RPE). We also obtained the joint automatic segmentation of the different retinal layers: from RNFL to ONL or inner retina (INNER); photoreceptors and RPE or outer retina (OUTER); all the retinal layers or the complete retina (RETINA).

Every scan was inspected by the same ophthalmologist (A.P.C) to detect segmentation errors and other issues, such as misalignments, decentration or motion artifacts. No manual adjustments were made. Because of the specular nomenclature of the macular grid, we represent the left eye data in the right eye format.

### Statistical Analysis

**Figure 1:**
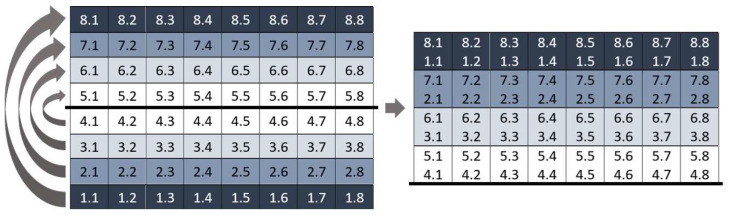
Corresponding cells of the 8 × 8 macular grid. All cells on the same-colored line in the superior and inferior hemispheres are corresponding cells. For example, cell 1.1 and cell 8.1 are corresponding cells.

All the statistical analyses were run using the SPSS software (version 26.0; SPSS Inc., Chicago, IL, USA). We assessed the normal distribution of all thickness values using the Kolmogorov–Smirnov test. We analyzed the thickness differences between the mean thickness of the 32 inferior cells and the mean thickness of the 32 superior cells of all the macular layers using Student’s *t*-test for paired samples. We also compared the thickness of each cell in the inferior hemisphere to the thickness of the corresponding cells in the superior hemisphere using Student’s *t*-test for paired samples (Figure 1). We expressed the results as the mean ± standard deviation (SD) of the thickness differences. Heatmaps depicting half the mean thickness difference between corresponding cells were plotted to highlight the inferior–superior asymmetry. Finally, we calculated the 2.5th and the 97.5th percentiles of the thickness differences in the 64 cells of all the macular layers. A *p* value < 0.05 was considered statistically significant.

## 3. Results

In total, 300 eyes of 300 subjects were included in this study, of which 152 were right eyes (51%) and 147 were left eyes (49%). The mean age of the men (50%) and women (50%) enrolled in this study was 49.78 ± 17.41 years (range 18–84). The mean axial length was 23.64 ± 0.90 mm (range 21.25–25.95). The mean keratometry was 44.14 ± 1.45 diopters for the steepest meridian and 43.25 ± 1.40 diopters for the flattest. The mean pachymetry was 530.40 ± 36.87 microns, and the mean adjusted IOP was 15.60 ± 2.94 mmHg.

When comparing the mean thickness values between hemispheres (Table 1, see below), we observed that the thickness of the OUTER and complete RETINA was significantly thicker in the superior than in the inferior hemisphere, whereas the thickness of the INNER retina was similar between hemispheres. The thickness of RNFL and OPL was significantly thicker in the inferior than in the superior hemisphere. Conversely, the thickness of IPL, ONL and RPE was significantly thinner in the inferior than in the superior hemisphere. The thickness of GCL and INL did not show any significant differences between hemispheres.

The results of the comparison of the thicknesses between the corresponding cells of the inferior and superior hemispheres of the different macular layers, and of the 2.5th and 97.5th percentiles of these thickness differences, are shown in Tables S1–S10.

**Figure 2:**
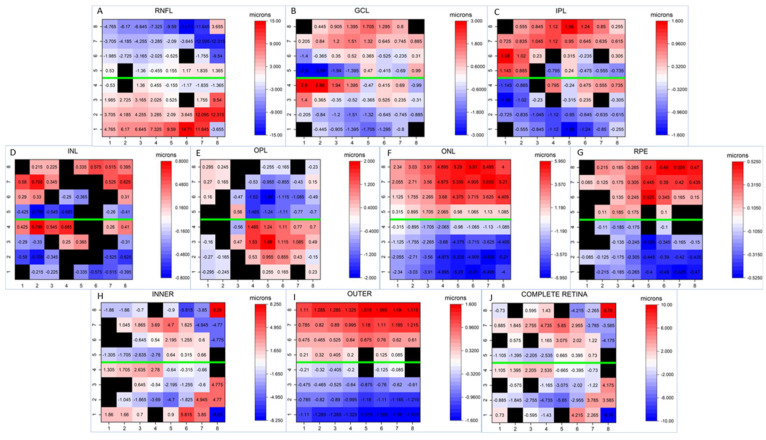
Asymmetry thickness heatmaps of the different retinal layers. Note that half the mean thickness differences between corresponding cells are plotted when significant differences are found. Red represents thickening, and blue depicts thinning. Corresponding cells with no statistically significant difference are shown in black. The horizontal midline is represented in green. All segmentations are represented as if all eyes were right eyes. Abbreviations: (**A**) RNFL: retinal nerve fiber layer; (**B**) GCL: ganglion cell layer; (**C**) IPL: inner plexiform layer; (**D**) INL: inner nuclear layer; (**E**) OPL: outer plexiform layer; (**F**) ONL: outer nuclear layer; (**G**) RPE: retinal pigmentary epithelium; (**H**) INNER: inner retina; (**I**) OUTER: outer retina; (**J**) RETINA: complete retina.

In order to better visually understand the differences between corresponding cells, Figure 2 provides heatmaps illustrating the asymmetry in thickness for each macular layer in relation to the horizontal midline. As depicted, significantly greater thicknesses were observed in most of the inferior cells on RNFL than in the corresponding superior cells, with larger differences in the peripheral cells than in the central cells. GCL and INL displayed a similar asymmetric pattern: significantly thicker cells were detected in most superior cells than in the corresponding inferior cells, but the temporal inferior cells near the horizontal midline were significantly thicker than their corresponding superior cells. However, significant differences were detected in fewer corresponding cells in INL than in GCL. IPL showed significantly thicker cells in the superior hemisphere, except in the central and nasally paracentral cells, where the pattern was the inverse. All the superior cells in ONL and most of the superior cells in RPE were significantly thicker than their corresponding inferior cells, while OPL had significantly higher thickness values in the inferonasal cells. We detected significantly thicker cells in the paracentral superonasal cells in the INNER and complete RETINA layers, while the peripheral superonasal cells and central superotemporal cells were significantly thinner than their corresponding cells on these layers. Finally, we observed that the OUTER layer was thicker in the superior hemisphere.

## 4. Discussion

The PPA was designed in 2011 as a novel tool for the diagnosis and follow-up of glaucoma [16]. This protocol is based on the asymmetric nature of this disease, and it establishes comparisons between the thickness value of a given cell in the inferior macular hemisphere and the thickness value of its corresponding superior cell in the same eye. The results are then displayed on a gray-colored asymmetric thickness map. Unlike the Early Treatment Diabetic Retinopathy Study (ETDRS) map, which is usually employed for macular explorations, the 8 × 8 PPA provides thickness values at 64 different points of the macula, is oriented along the fovea–disk axis and respects the horizontal midline. Therefore, this protocol seems more appropriate to analyze macular thickness asymmetries than the ETDRS.

Research works into the presence of intraocular physiological macular thickness asymmetry are limited [17,18,19,20,21]. According to previous studies [17,18,19,20,21], the results of the present study showed that the thickness asymmetries between the superior and inferior hemispheres of the macula are present in healthy eyes. This finding highlights the need to develop a normative database that allows improvements to be made to the interpretation of tomography images and to identify incipient pathological asymmetries of macular thicknesses secondary to glaucoma [8] or to other retinal or neuropsychiatric diseases [1,2,3,4,5,6].

Altemir et al. [22] established that the normal range of ocular thickness asymmetries corresponds to the 95th central percentiles values that, in turn, correspond to the values between the 2.5th and 97.5th percentiles of the thickness asymmetry detected in a healthy population with normal distribution. Thus asymmetric thickness values below the 2.5th percentile or above the 97.5th percentile should be considered likely to be pathological asymmetric thickness values.

A previous study [17] reported similar mean thickness values of the 32 cells of the superior macular hemisphere and of the 32 cells of the inferior macular hemisphere of the total retina to the results of this study when analyzing a healthy Caucasian adult population. However, conversely to our results, these authors did not find any significant statistical thickness differences between hemispheres, likely due to their smaller sample size (n = 105) than herein (n = 300). We detected that the standard deviation of the mean thickness values, which represents the inter-individual variability of macular thickness, was lower when analyzing individual cells than when clustering several cells (mean ± SD thickness). This finding agrees with other previous studies [17,23]. Therefore, the analysis of the thickness asymmetry for each couple of corresponding cells of the different macular layers could constitute a more sensitive test than those that analyze sectors, like the peripapillary retinal nerve layer thickness analysis or the macular ganglion cell layer analysis [15]. Furthermore, a previous study [21] demonstrated that age, gender and axial length have less impact on the intraocular retinal thickness asymmetry than on the quantitative retinal thickness values.

Two previous research works [19,20] also employed the PPA of the Spectralis to analyze the presence of the thickness asymmetries of the total retina in each couple of corresponding cells of the macular grid. One of them [20] showed significant thickness asymmetries in some of the peripheral and nasal cells of the macular grid, while our results revealed statistically significant differences in most of the couples of the corresponding cells of the complete RETINA. These differences could be explained by the higher statistical power in our study due to its bigger sample size.

We can also see that the absolute values of the thickness asymmetries of the complete RETINA in our study and other previous studies [19,20] differ, likely due to the distinct sample populations’ demographic and ophthalmological characteristics. So the normative database shown in our study should be employed mainly to analyze Caucasian populations.

Through the thickness asymmetry analysis of each couple of the corresponding cells between the inferior and superior hemispheres on different macular layers, we observed that the magnitude of the thickness asymmetries was not homogeneously distributed. We found larger significant intraocular thickness asymmetries on RNFL and ONL, while the RPE displayed a minimal significant thickness asymmetry between hemispheres. Other authors [21] also indicate more intraocular variations in RNFL thickness than on GCL and IPL when analyzing the physiological intraocular thickness asymmetries of the inner retinal layers. Conversely to all these findings, a previous study [17] did not show any significant asymmetries thickness on GCL, likely because these authors analyzed grouped cells instead of individual ones.

As we can see, most of the thickness asymmetry values included within the normal ranges (Tables S1–S10) are lower than 30 microns. Thus, the PPA should be able to detect pathological thickness asymmetries because it is able to represent differences of up to 30 microns [16]. However, we also note that the 2.5th and 97.5th percentile values of the thickness asymmetries are larger than 30 microns in some couples of the corresponding cells on RNFL, and in three couples of the corresponding cells on ONL that could be considered physiological. Another remarkable finding in our study is that GCL did not show either 2.5th or 97.5th percentile values of thickness asymmetries larger than 30 micros. Furthermore, a previous study [21] demonstrated less physiological variation in the intraocular thickness asymmetry for GCL than for RNFL. This means that the analysis of the intraocular thickness asymmetries of GCL using the PPA could constitute the most sensitive and specific test for the screening and follow-up of glaucoma.

### Limitations

Our study has some limitations. First of all, the PPA is only able to detect intraocular macular thickness differences up to 30 microns, while physiological thickness asymmetries can exceed 30 in some macular layers. As we included only Caucasian adult subjects and we excluded eyes with high refractive errors, this normative database should be employed to analyze populations with the same characteristics. Finally, one may argue that the sample size of the studied group of subjects is relatively small (n = 300). However, we should keep in mind that other commercial normative databases have a smaller sample size than ours [24].

## 5. Conclusions

This study demonstrates that there are physiological macular thickness asymmetries in healthy eyes between the superior and inferior hemispheres. This study provides the first normative database of the thickness asymmetries on each macular layer to improve the classification of OCT images into normal or pathological findings when analyzing macular thickness asymmetries.

## Figures and Tables

**Table 1:** Analysis of the retinal layer thickness between the superior and inferior hemispheres of the macula using Student’s test for paired samples (*n* = 300). The superior hemisphere was considered the mean of the 32 superior cells and the inferior hemisphere was taken as the mean of the 32 inferior cells of the 8 × 8 grid. Abbreviations: RNFL: retinal nerve fiber layer; GCL: ganglion cell layer; IPL: inner plexiform layer; INL: inner nuclear layer; OPL: outer plexiform layer; ONL: outer nuclear layer; RPE: retinal pigmentary epithelium; INNER: inner retina; OUTER: outer retina; RETINA: complete retina.

Layer	Hemisphere	Mean ± SD	*p* Value
RNFL	Superior	38.76 ± 25.03	<0.001
	Inferior	46.33 ± 30.36	
GCL	Superior	33.05 ± 10.34	0.333
	Inferior	32.89 ± 10.88	
IPL	Superior	27.79 ± 8.21	<0.001
	Inferior	26.77 ± 8.66	
INL	Superior	31.61 ± 5.88	0.054
	Inferior	31.43 ± 6.39	
OPL	Superior	26.20 ± 5.12	<0.001
	Inferior	27.14 ± 6.26	
ONL	Superior	55.54 ± 11.08	<0.001
	Inferior	51.98 ± 11.74	
RPE	Superior	13.03 ± 2.49	<0.001
	Inferior	12.57 ± 2.22	
INNER	Superior	215.92 ± 33.27	0.066
	Inferior	216.62 ± 33.58	
OUTER	Superior	78.61 ± 3.65	<0.001
	Inferior	77.00 ± 3.81	
RETINA	Superior	294.53 ± 34.46	<0.001
	Inferior	293.61 ± 34.96	

## Data Availability

Our data are shown in the main text or as Supplementary Materials.

## Supplementary Materials

The following supporting information can be downloaded at: https://www.mdpi.com/article/10.3390/jcm12247609/s1, Table S1. Mean ± SD of the thickness difference between corresponding cells of the inferior and superior hemispheres of the retinal nerve fiber layer (RNFL) and its statistical significance (*p* value) (Student’s test for paired samples). Positive values indicate that thicker thicknesses are detected in the inferior cells than in its corresponding cells in the superior hemisphere. Negative values indicate that inferior cell show thinner thicknesses than its corresponding cells in the superior hemisphere. 2.5th and 97.5th percentiles of the asymmetry thickness in each cell of the 8 × 8 grid for the RNFL are also shown; Table S2. Table S2. Mean ± SD of the thickness difference between corresponding cells of the inferior and superior hemispheres of the ganglion cell layer (GCL) and its statistical significance (*p* value) (Student’s test for paired samples). Positive values indicate that thicker thicknesses are detected in the inferior cells than in its corresponding cells in the superior hemisphere. Negative values indicate that inferior cell show thinner thicknesses than its corresponding cells in the superior hemisphere. 2.5th and 97.5th percentiles of the asymmetry thickness in each cell of the 8 × 8 grid for the GCL are also shown; Table S3. Mean ± SD of the thickness difference between corresponding cells of the inferior and superior hemispheres of the inner plexiform layer (IPL) and its statistical significance (*p* value) (Student’s test for paired samples). Positive values indicate that thicker thicknesses are detected in the inferior cells than in its corresponding cells in the superior hemisphere. Negative values indicate that inferior cell show thinner thicknesses than its corresponding cells in the superior hemisphere. 2.5th and 97.5th percentiles of the asymmetry thickness in each cell of the 8 × 8 grid for the IPL are also shown; Table S4. Mean ± SD of the thickness difference between corresponding cells of the inferior and superior hemispheres of the inner nuclear layer (INL) and its statistical significance (*p* value) (Student’s test for paired samples). Positive values indicate that thicker thicknesses are detected in the inferior cells than in its corresponding cells in the superior hemisphere. Negative values indicate that inferior cell show thinner thicknesses than its corresponding cells in the superior hemisphere. 2.5th and 97.5th percentiles of the asymmetry thickness in each cell of the 8x8 grid for the INL are also shown; Table S5. Mean ± SD of the thickness difference between corresponding cells of the inferior and superior hemispheres of the outer plexiform layer (OPL) and its statistical significance (p value) (Student’s test for paired samples). Positive values indicate that thicker thicknesses are detected in the inferior cells than in its corresponding cells in the superior hemisphere. Negative values indicate that inferior cell show thinner thicknesses than its corresponding cells in the superior hemisphere. 2.5th and 97.5th percentiles of the asymmetry thickness in each cell of the 8 × 8 grid for the OPL are also shown; Table S6. Mean ± SD of the thickness difference between corresponding cells of the inferior and superior hemispheres of the outer nuclear layer (ONL) and its statistical significance (*p* value) (Student’s test for paired samples). Positive values indicate that thicker thicknesses are detected in the inferior cells than in its corresponding cells in the superior hemisphere. Negative values indicate that inferior cell show thinner thicknesses than its corresponding cells in the superior hemisphere. 2.5th and 97.5th percentiles of the asymmetry thickness in each cell of the 8 × 8 grid for the ONL are also shown; Table S7. Mean ± SD of the thickness difference between corresponding cells of the inferior and superior hemispheres of the retinal pigmentary epithelium (RPE) and its statistical significance (*p* value) (Student’s test for paired samples). Positive values indicate that thicker thicknesses are detected in the inferior cells than in its corresponding cells in the superior hemisphere. Negative values indicate that inferior cell show thinner thicknesses than its corresponding cells in the superior hemisphere. 2.5th and 97.5th percentiles of the asymmetry thickness in each cell of the 8 × 8 grid for the RPE are also shown; Table S8. Mean ± SD of the thickness difference between corresponding cells of the inferior and superior hemispheres of the inner retina (INNER) and its statistical significance (*p* value) (Student’s test for paired samples). Positive values indicate that thicker thicknesses are detected in the inferior cells than in its corresponding cells in the superior hemisphere. Negative values indicate that inferior cell show thinner thicknesses than its corresponding cells in the superior hemisphere. 2.5th and 97.5th percentiles of the asymmetry thickness in each cell of the 8 × 8 grid for the INNER are also shown: Table S9. Mean ± SD of the thickness difference between corresponding cells of the inferior and superior hemispheres of the outer retina (OUTER) and its statistical significance (*p* value) (Student’s test for paired samples). Positive values indicate that thicker thicknesses are detected in the inferior cells than in its corresponding cells in the superior hemisphere. Negative values indicate that inferior cell show thinner thicknesses than its corresponding cells in the superior hemisphere. 2.5th and 97.5th percentiles of the asymmetry thickness in each cell of the 8 × 8 grid for the OUTER are also shown; Table S10. Mean ± SD of the thickness difference between corresponding cells of the inferior and superior hemispheres of the complete retina (RETINA) and its statistical significance (*p* value) (Student’s test for paired samples). Positive values indicate that thicker thicknesses are detected in the inferior cells than in its corresponding cells in the superior hemisphere. Negative values indicate that inferior cell show thinner thicknesses than its corresponding cells in the superior hemisphere. 2.5th and 97.5th percentiles of the asymmetry thickness in each cell of the 8 × 8 grid for the RETINA are also shown.
