# Supplementary material for: Normative Database of the Superior–Inferior Thickness Asymmetry for All Inner and Outer Macular Layers of Adults for the Posterior Pole Algorithm of the Spectralis SD-OCT
Source: J Clin Med. 2023 Dec 11;12(24):7609. doi: 10.3390/jcm12247609 (PMC10743748; doi:10.3390/jcm12247609)
Supplement: Supplementary file 1 [file jcm-12-07609-s001.zip › jcm-2729079-supplementary.pdf]

Table S1. Mean ± SD of the thickness difference between corresponding cells of the inferior and superior hemispheres of the retinal nerve fiber layer (RNFL) and its statistical significance (p value) (Student's test for paired samples). Positive values indicate that thicker thicknesses are detected in the inferior cells than in its corresponding cells in the superior hemisphere. Negative values indicate that inferior cell show thinner thicknesses than its corresponding cells in the superior hemisphere. 2.5^{th} *and* 97.5^{th} *percentiles of the asymmetry thickness in each cell of the 8x8 grid for the RNFL are also shown.*

RNFL	1.1-8.1	1.2-8.2	1.3-8.3	1.4-8.4	1.5-8.5	1.6-8.6	1.7-8.7	1.8-8.8
Mean ± SD	9.53 ± 5.99	12.34 ± 7.46	13.29 ± 8.11	14.65 ± 9.84	19.18 ± 13.46	29.42 ± 16.17	23.29 ± 20.36	-7.31 ± 22.21
p value	**<0.001**	**<0.001**	**<0.001**	**<0.001**	**<0.001**	**<0.001**	**<0.001**	**<0.001**
p2.5	-2.00	-1.47	-0.47	-2.47	-6.00	-3.47	-16.48	-48.00
p97.5	21.00	28.47	33.00	38.00	50.00	59.47	61.47	38.00
	2.1-7.1	2.2-7.2	2.3-7.3	2.4-7.4	2.5-7.5	2.6-7.6	2.7-7.7	2.8-7.8
Mean ± SD	7.41 ± 4.95	8.37 ± 4.82	8.51 ± 5.04	6.57 ± 5.04	4.18 ± 6.04	7.29 ± 9.79	24.19 ± 16.47	24.63 ± 21.46
p value	**<0.001**	**<0.001**	**<0.001**	**<0.001**	**<0.001**	**<0.001**	**<0.001**	**<0.001**
p2.5	-1.47	-1.00	-2.47	-4.00	-8.00	-13.00	-5.95	-22.00
p97.5	20.00	18.00	18.00	17.00	15.47	32.47	56.42	63.47
	3.1-6.1	3.2-6.2	3.3-6.3	3.4-6.4	3.5-6.5	3.6-6.6	3.7-6.7	3.8-6.8
Mean ± SD	3.97 ± 3.38	5.45 ± 3.72	6.33 ± 3.76	4.05 ± 3.71	1.05 ± 4.15	-7.54 ± 6.90	3.51 ± 7.76	19.08 ± 16.18
p value	**<0.001**	**<0.001**	**<0.001**	**<0.001**	**<0.001**	0.505	**<0.001**	**<0.001**
p2.5	-1.00	0.00	-1.47	-5.00	-8.00	-10.00	-13.00	-11.00
p97.5	11.00	13.00	14.47	11.47	9.00	9.00	18.95	52.95
	4.1-5.1	4.2-5.2	4.3-5.3	4.4-5.4	4.5-5.5	4.6-5.6	4.7-5.7	4.8-5.8
Mean ± SD	-1.06 ± 2.95	-0.39 ± 3.81	2.72 ± 2.68	0.91 ± 1.67	-0.31 ± 1.96	-2.34 ± 3.20	-3.67 ± 4.09	-2.73 ± 9.75
p value	**<0.001**	0.080	**<0.001**	**<0.001**	**0.007**	**<0.001**	**<0.001**	**<0.001**
p2.5	-6.00	-7.00	-3.00	-3.00	-4.00	-9.47	-13.00	-21.00
p97.5	5.00	6.00	7.00	4.00	3.00	4.47	5.47	20.47

Table S2. Mean ± SD of the thickness difference between corresponding cells of the inferior and superior hemispheres of the ganglion cell layer (GCL) and its statistical significance (p value) (Student's test for paired samples). Positive values indicate that thicker thicknesses are detected in the inferior cells than in its corresponding cells in the superior hemisphere. Negative values indicate that inferior cell show thinner thicknesses than its corresponding cells in the superior hemisphere. 2.5th and 97.5th percentiles of the asymmetry thickness in each cell of the 8x8 grid for the GCL are also shown.

GCL	1.1-8.1	1.2-8.2	1.3-8.3	1.4-8.4	1.5-8.5	1.6-8.6	1.7-8.7	1.8-8.8
Mean ± SD	-0.27 ± 2.51	-0.89 ± 2.44	-1.81 ± 2.56	-2.79 ± 2.71	-3.41 ± 2.72	-2.59 ± 2.82	-1.60 ± 3.01	-0.31 ± 2.95
p value	0.067	**<0.001**	**<0.001**	**<0.001**	**<0.001**	**<0.001**	**<0.001**	0.075
p2.5	-5.00	-6.00	-6.00	-8.00	-8.00	-8.00	-7.00	-7.00
p97.5	4.00	3.00	3.00	3.00	2.00	3.00	5.00	6.00
	2.1-7.1	2.2-7.2	2.3-7.3	2.4-7.4	2.5-7.5	2.6-7.6	2.7-7.7	2.8-7.8
Mean ± SD	-0.41 ± 2.51	-1.68 ± 2.71	-2.40 ± 3.19	-3.02 ± 3.62	-2.64 ± 3.45	-1.29 ± 2.97	-1.49 ± 3.00	-1.77 ± 2.85
p value	**0.005**	**<0.001**	**<0.001**	**<0.001**	**<0.001**	**<0.001**	**<0.001**	**<0.001**
p2.5	-6.00	-7.00	-9.00	-9.47	-9.00	-6.47	-7.00	-8.00
p97.5	4.00	3.00	3.00	3.00	4.00	5.00	5.47	4.00
	3.1-6.1	3.2-6.2	3.3-6.3	3.4-6.4	3.5-6.5	3.6-6.6	3.7-6.7	3.8-6.8
Mean ± SD	2.80 ± 2.93	0.73 ± 2.96	-0.70 ± 3.65	-1.04 ± 3.34	-0.73 ± 3.15	1.05 ± 3.62	0.47 ± 2.90	-0.62 ± 2.83
p value	**<0.001**	**<0.001**	**0.001**	**<0.001**	**<0.001**	**<0.001**	**0.006**	**<0.001**
p2.5	-4.47	-6.00	-8.00	-7.47	-7.47	-6.00	-5.00	-6.00
p97.5	8.00	7.00	6.47	6.00	5.00	9.00	7.47	6.00
	4.1-5.1	4.2-5.2	4.3-5.3	4.4-5.4	4.5-5.5	4.6-5.6	4.7-5.7	4.8-5.8
Mean ± SD	5.80 ± 3.31	5.72 ± 3.60	3.88 ± 2.81	2.79 ± 6.16	-0.94 ± 5.56	0.83 ± 2.39	1.38 ± 2.23	-1.98 ± 2.35
p value	**<0.001**	**<0.001**	**<0.001**	**<0.001**	**0.004**	**<0.001**	**<0.001**	**<0.001**
p2.5	-2.00	-2.00	-1.47	-11.00	-11.47	-3.47	-3.00	-2.47
p97.5	12.00	12.00	10.00	14.00	11.00	5.47	6.00	7.00

Table S3. Mean ± SD of the thickness difference between corresponding cells of the inferior and superior hemispheres of the inner plexiform layer (IPL) and its statistical significance (p value) (Student's test for paired samples). Positive values indicate that thicker thicknesses are detected in the inferior cells than in its corresponding cells in the superior hemisphere. Negative values indicate that inferior cell show thinner thicknesses than its corresponding cells in the superior hemisphere. 2.5th and 97.5th percentiles of the asymmetry thickness in each cell of the 8x8 grid for the IPL are also shown.

IPL	1.1-8.1	1.2-8.2	1.3-8.3	1.4-8.4	1.5-8.5	1.6-8.6	1.7-8.7	1.8-8.8
Mean ± SD	-2.33 ± 2.65	-1.11 ± 2.53	-1.69 ± 2.85	-2.24 ± 2.72	-3.12 ± 2.91	-2.48 ± 3.08	-1.70 ± 3.45	-0.51 ± 4.19
p value	0.128	**<0.001**	**<0.001**	**<0.001**	**<0.001**	**<0.001**	**<0.001**	**0.037**
p2.5	-6.00	-6.00	-8.47	-8.00	-8.00	-8.00	-8.47	-9.00
p97.5	5.47	4.00	3.00	2.00	2.47	4.47	6.00	7.47
	2.1-7.1	2.2-7.2	2.3-7.3	2.4-7.4	2.5-7.5	2.6-7.6	2.7-7.7	2.8-7.8
Mean ± SD	-1.45 ± 2.79	-1.67 ± 2.65	-2.09 ± 2.93	-2.24 ± 3.24	-1.90 ± 2.97	-1.29 ± 2.55	-1.27 ± 2.92	-1.23 ± 3.47
p value	**<0.001**	**<0.001**	**<0.001**	**<0.001**	**<0.001**	**<0.001**	**<0.001**	**<0.001**
p2.5	-7.00	-7.47	-8.47	-8.00	-7.00	-6.00	-7.00	-8.00
p97.5	4.00	3.00	4.00	4.00	4.00	4.00	6.00	7.00
	3.1-6.1	3.2-6.2	3.3-6.3	3.4-6.4	3.5-6.5	3.6-6.6	3.7-6.7	3.8-6.8
Mean ± SD	-3.16 ± 2.94	-2.04 ± 0.92	-0.46 ± 3.11	-0.30 ± 2.69	-0.63 ±2.93	0.47 ± 2.90	-0.70 ± 2.41	-0.61 ± 2.94
p value	**<0.001**	**<0.001**	**0.011**	0.054	**<0.001**	**0.005**	0.615	**<0.001**
p2.5	-8.47	-8.00	-6.00	-5.47	-6.00	-5.00	-4.00	-6.00
p97.5	3.00	4.00	5.00	5.00	5.00	6.95	5.00	6.00
	4.1-5.1	4.2-5.2	4.3-5.3	4.4-5.4	4.5-5.5	4.6-5.6	4.7-5.7	4.8-5.8
Mean ± SD	-2.29 ± 2.72	-1.77 ± 2.54	-0.183 ± 2.27	1.59 ± 3.63	-0.48 ± 3.47	0.95 ± 1.98	1.11 ± 1.92	1.47 ± 2.82
p value	**<0.001**	**<0.001**	0.163	**<0.001**	**0.017**	**<0.001**	**<0.001**	**<0.001**
p2.5	-7.47	-7.00	-5.00	-5.47	-8.00	-3.00	-3.00	-4.00
p97.5	3.00	3.00	4.00	8.47	6.00	5.00	5.00	8.00

Table S4. Mean ± SD of the thickness difference between corresponding cells of the inferior and superior hemispheres of the inner nuclear layer (INL) and its statistical significance (p value) (Student's test for paired samples). Positive values indicate that thicker thicknesses are detected in the inferior cells than in its corresponding cells in the superior hemisphere. Negative values indicate that inferior cell show thinner thicknesses than its corresponding cells in the superior hemisphere. 2.5th and 97.5th percentiles of the asymmetry thickness in each cell of the 8x8 grid for the INL are also shown.

INL	1.1-8.1	1.2-8.2	1.3-8.3	1.4-8.4	1.5-8.5	1.6-8.6	1.7-8.7	1.8-8.8
Mean ± SD	-0.20 ± 3.77	-0.43 ± 3.16	-0.45 ± 3.31	-0.46 ± 4.07	-0.67 ± 4.23	-1.15 ± 4.09	-1.03 ± 4.34	-0.79 ± 5.38
p value	0.927	**0.018**	**0.019**	0.050	**0.006**	**<0.001**	**<0.001**	**0.011**
p2.5	-6.00	-6.00	-6.00	-7.00	-7.00	-7.47	-8.47	-11.00
p97.5	11.95	7.47	7.47	9.90	9.47	8.00	8.47	11.47
	2.1-7.1	2.2-7.2	2.3-7.3	2.4-7.4	2.5-7.5	2.6-7.6	2.7-7.7	2.8-7.8
Mean ± SD	-1.16 ± 2.82	-1.41 ± 2.86	-0.69 ± 3.30	+0.60 ± 3.82	-0.22 ± 3.40	-0.26 ± 2.93	-1.05 ± 2.95	-1.25 ± 3.71
p value	**<0.001**	**<0.001**	**<0.001**	0.786	0.256	0.125	**<0.001**	**<0.001**
p2.5	-6.00	-6.47	-6.00	-5.00	-6.00	-5.00	-6.00	-8.00
p97.5	5.00	5.00	5.00	6.47	8.00	6.47	5.00	8.00
	3.1-6.1	3.2-6.2	3.3-6.3	3.4-6.4	3.5-6.5	3.6-6.6	3.7-6.7	3.8-6.8
Mean ± SD	-0.58 ± 2.91	-0.66 ± 2.57	-0.30 ± 2.93	0.50 ± 3.10	0.73 ± 3.70	0.01 ± 3.47	0.00 ± 2.64	-0.62 ± 3.14
p value	**0.001**	**<0.001**	0.077	**0.006**	**0.001**	0.974	1.000	**0.001**
p2.5	-7.00	-6.00	-6.00	-5.47	-6.47	-6.00	-5.00	-6.00
p97.5	4.00	4.47	5.47	7.00	9.00	7.00	6.00	6.47
	4.1-5.1	4.2-5.2	4.3-5.3	4.4-5.4	4.5-5.5	4.6-5.6	4.7-5.7	4.8-5.8
Mean ± SD	0.85 ± 2.38	1.59 ± 2.38	1.09 ± 2.80	1.37 ± 4.23	-0.04 ± 4.21	-0.06 ± 3.38	0.52 ± 3.00	0.82 ± 3.18
p value	**<0.001**	**<0.001**	**<0.001**	**<0.001**	0.870	0.772	**0.003**	**<0.001**
p2.5	-4.00	-3.00	-4.47	-7.47	-9.47	-6.47	-5.00	-7.00
p97.5	6.00	6.00	7.00	10.00	8.00	7.47	7.00	7.00

Table S5. Mean ± SD of the thickness difference between corresponding cells of the inferior and superior hemispheres of the outer plexiform layer (OPL) and its statistical significance (p value) (Student's test for paired samples). Positive values indicate that thicker thicknesses are detected in the inferior cells than in its corresponding cells in the superior hemisphere. Negative values indicate that inferior cell show thinner thicknesses than its corresponding cells in the superior hemisphere. 2.5th and 97.5th percentiles of the asymmetry thickness in each cell of the 8x8 grid for the OPL are also shown.

OPL	1.1-8.1	1.2-8.2	1.3-8.3	1.4-8.4	1.5-8.5	1.6-8.6	1.7-8.7	1.8-8.8
Mean ± SD	-0.59 ± 2.19	-0.49 ± 2.15	-0.16 ± 2.30	0.25 ± 2.53	0.51 ± 2.45	0.33 ± 2.39	-0.17 ± 2.19	0.46 ± 2.27
p value	**<0.001**	**<0.001**	0.230	0.092	**<0.001**	**0.016**	0.172	**<0.001**
p2.5	-4.47	-4.00	-5.00	-5.47	-5.00	-5.95	-5.00	-4.00
p97.5	4.00	4.00	4.00	4.00	6.00	5.00	4.47	5.00
	2.1-7.1	2.2-7.2	2.3-7.3	2.4-7.4	2.5-7.5	2.6-7.6	2.7-7.7	2.8-7.8
Mean ± SD	-0.54 ± 2.12	-0.33 ± 2.33	0.70 ± 2.70	1.06 ± 3.42	1.91 ± 3.96	1.71 ± 3.54	0.86 ± 2.65	-0.30 ± 2.50
p value	**<0.001**	**0.014**	0.654	**<0.001**	**<0.001**	**<0.001**	**<0.001**	**0.036**
p2.5	-4.00	-5.00	-7.00	-6.00	-6.00	-5.47	-4.47	-5.00
p97.5	3.00	3.47	5.00	8.00	11.00	9.00	6.47	6.00
	3.1-6.1	3.2-6.2	3.3-6.3	3.4-6.4	3.5-6.5	3.6-6.6	3.7-6.7	3.8-6.8
Mean ± SD	-0.32 ± 1.96	-0.21 ± 2.71	0.94 ± 4.02	3.06 ±7.58	3.96 ± 8.93	2.23 ± 5.85	2.17 ± 3.64	0.98 ± 2.75
p value	**0.005**	0.174	**<0.001**	**<0.001**	**<0.001**	**<0.001**	**<0.001**	**<0.001**
p2.5	-4.00	-7.00	-6.00	-11.47	-13.47	-8.00	-5.00	-4.00
p97.5	3.00	5.00	10.00	22.47	24.00	16.00	11.00	7.00
	4.1-5.1	4.2-5.2	4.3-5.3	4.4-5.4	4.5-5.5	4.6-5.6	4.7-5.7	4.8-5.8
Mean ± SD	0.03 ± 1.70	0.02 ± 2.33	-1.12 ± 4.95	2.97 ± 10.89	2.48 ± 12.51	2.22 ± 6.54	1.54 ± 3.29	1.40 ± 2.66
p value	0.786	0.882	**<0.001**	**<0.001**	**0.001**	**<0.001**	**<0.001**	**<0.001**
p2.5	-3.00	-4.00	-9.00	-19.00	-23.00	-10.47	-4.47	-4.00
p97.5	3.00	5.00	15.47	30.00	31.47	18.95	10.00	7.00

Table S6. Mean ± SD of the thickness difference between corresponding cells of the inferior and superior hemispheres of the outer nuclear layer (ONL) and its statistical significance (p value) (Student's test for paired samples). Positive values indicate that thicker thicknesses are detected in the inferior cells than in its corresponding cells in the superior hemisphere. Negative values indicate that inferior cell show thinner thicknesses than its corresponding cells in the superior hemisphere. 2.5th and 97.5th percentiles of the asymmetry thickness in each cell of the 8x8 grid for the ONL are also shown.

ONL	1.1-8.1	1.2-8.2	1.3-8.3	1.4-8.4	1.5-8.5	1.6-8.6	1.7-8.7	1.8-8.8
Mean ± SD	-4.68 ± 3.27	-6.06 ± 3.22	-7.82 ± 3.53	-9.79 ± 4.14	-10.58 ± 4.60	-11.82 ± 4.83	-10.99 ± 5.43	-8.00 ± 6.57
p value	**<0.001**	**<0.001**	**<0.001**	**<0.001**	**<0.001**	**<0.001**	**<0.001**	**<0.001**
p2.5	-12.00	-13.00	-14.47	-18.00	-20.48	-20.48	-20.00	-19.00
p97.5	1.00	0.00	-0.53	-2.00	-2.00	-3.00	-2.00	1.00
	2.1-7.1	2.2-7.2	2.3-7.3	2.4-7.4	2.5-7.5	2.6-7.6	2.7-7.7	2.8-7.8
Mean ± SD	-4.11 ± 2.63	-5.42 ± 3.02	-7.12 ± 4.05	-9.75 ± 5.21	-10.67 ± 5.48	-9.81 ± 5.02	-11.31 ± 4.80	-10.42 ± 5.36
p value	**<0.001**	**<0.001**	**<0.001**	**<0.001**	**<0.001**	**<0.001**	**<0.001**	**<0.001**
p2.5	-9.47	-12.00	-15.47	-21.00	-22.47	-20.00	-20.00	-21.00
p97.5	1.00	0.00	2.00	1.47	-1.00	0.00	-1.00	0.00
	3.1-6.1	3.2-6.2	3.3-6.3	3.4-6.4	3.5-6.5	3.6-6.6	3.7-6.7	3.8-6.8
Mean ± SD	-2.25 ± 2.78	-3.51 ± 3.38	-4.53 ± 5.28	-7.36 ± 8.68	-8.75 ± 10.19	-7.43 ± 6.95	-7.25 ± 4.65	-8.81 ± 5.00
p value	**<0.001**	**<0.001**	**<0.001**	**<0.001**	**<0.001**	**<0.001**	**<0.001**	**<0.001**
p2.5	-9.00	-9.47	-18.00	-28.47	-32.42	-23.47	-17.00	-20.00
p97.5	3.47	4.00	7.47	9.95	14.00	6.47	3.00	2.00
	4.1-5.1	4.2-5.2	4.3-5.3	4.4-5.4	4.5-5.5	4.6-5.6	4.7-5.7	4.8-5.8
Mean ± SD	-0.63 ± 2.13	-1.79 ± 2.52	-3.41 ± 5.69	-4.13 ± 11.98	-1.96 ± 13.51	-2.13 ± 7.15	-2.26 ± 3.63	-2.17 ± 4.25
p value	**<0.001**	**<0.001**	**<0.001**	**<0.001**	**0.012**	**<0.001**	**<0.001**	**<0.001**
p2.5	-5.00	-7.00	-19.48	-33.47	-32.00	-20.00	-10.00	-10.47
p97.5	4.00	3.47	8.00	20.00	26.00	11.00	4.00	6.00

Table S7. Mean ± SD of the thickness difference between corresponding cells of the inferior and superior hemispheres of the retinal pigmentary epithelium (RPE) and its statistical significance (p value) (Student's test for paired samples). Positive values indicate that thicker thicknesses are detected in the inferior cells than in its corresponding cells in the superior hemisphere. Negative values indicate that inferior cell show thinner thicknesses than its corresponding cells in the superior hemisphere. 2.5th and 97.5th percentiles of the asymmetry thickness in each cell of the 8x8 grid for the RPE are also shown.

RPE	1.1-8.1	1.2-8.2	1.3-8.3	1.4-8.4	1.5-8.5	1.6-8.6	1.7-8.7	1.8-8.8
Mean ± SD	-0.15 ± 2.87	-0.43 ± 2.19	-0.37 ± 1.70	-0.53 ± 1.58	-0.80 ± 1.94	-0.98 ± 1.90	-1.01 ± 1.75	-0.94 ± 2.38
p value	0.356	**0.001**	**<0.001**	**<0.001**	**<0.001**	**<0.001**	**<0.001**	**<0.001**
p2.5	-3.00	-3.00	-2.47	-3.00	-3.47	-4.00	-4.00	-4.47
p97.5	2.00	2.00	2.00	2.00	2.00	2.47	2.00	5.47
	2.1-7.1	2.2-7.2	2.3-7.3	2.4-7.4	2.5-7.5	2.6-7.6	2.7-7.7	2.8-7.8
Mean ± SD	-0.17 ± 1.23	-0.25 ± 1.24	-0.35 ± 1.06	-0.61 ± 1.16	-0.89 ± 1.40	-0.78 ± 1.78	-0.84 ± 1.41	-0.87 ± 2.55
p value	**0.020**	**0.001**	**<0.001**	**<0.001**	**<0.001**	**<0.001**	**<0.001**	**<0.001**
p2.5	-2.47	-2.00	-2.00	-3.00	-3.47	-3.00	-3.00	-6.00
p97.5	2.00	2.00	2.00	2.00	1.00	2.00	2.00	3.00
	3.1-6.1	3.2-6.2	3.3-6.3	3.4-6.4	3.5-6.5	3.6-6.6	3.7-6.7	3.8-6.8
Mean ± SD	-0.63 ± 1.22	-0.14 ± 1.27	-0.27 ± 1.15	-0.49 ± 1.13	-1.05 ± 1.81	-0.69 ± 1.99	-0.33 ± 1.72	-0.30 ± 3.35
p value	0.369	0.063	**<0.001**	**<0.001**	**<0.001**	**<0.001**	**0.001**	0.118
p2.5	-2.00	-2.00	-2.00	-3.00	-3.00	-3.00	-3.00	-4.00
p97.5	2.00	2.00	2.00	2.00	2.00	1.00	3.00	4.00
	4.1-5.1	4.2-5.2	4.3-5.3	4.4-5.4	4.5-5.5	4.6-5.6	4.7-5.7	4.8-5.8
Mean ± SD	-0.10 ± 1.07	-0.22 ± 0.96	-0.37 ± 1.55	-0.35 ± 2.06	-0.13 ± 1.52	-0.20 ± 1.10	-0.05 ± 1.17	-0.02 ± 1.86
p value	0.095	**<0.001**	**<0.001**	**0.004**	0.149	**0.002**	0.460	0.877
p2.5	-1.00	-2.00	-2.00	-3.00	-2.47	-2.00	-2.00	-2.00
p97.5	1.00	1.00	2.00	2.00	3.00	2.00	2.00	3.00

Table S8. Mean ± SD of the thickness difference between corresponding cells of the inferior and superior hemispheres of the inner retina (INNER) and its statistical significance (p value) (Student's test for paired samples). Positive values indicate that thicker thicknesses are detected in the inferior cells than in its corresponding cells in the superior hemisphere. Negative values indicate that inferior cell show thinner thicknesses than its corresponding cells in the superior hemisphere. 2.5th and 97.5th percentiles of the asymmetry thickness in each cell of the 8x8 grid for the INNER are also shown.

INNER	1.1-8.1	1.2-8.2	1.3-8.3	1.4-8.4	1.5-8.5	1.6-8.6	1.7-8.7	1.8-8.8
Mean ± SD	3.73 ± 9.03	3.32 ± 9.39	1.40 ± 9.60	-0.21 ± 10.91	1.80 ± 13.23	11.63 ± 14.96	7.70 ± 17.91	-16.50 ± 20.15
p value	**<0.001**	**<0.001**	**0.012**	0.739	**0.019**	**<0.001**	**<0.001**	**<0.001**
p2.5	-13.47	-15.47	-18.48	-20.00	-22.47	-15.95	-29.47	-53.00
p97.5	20.47	23.47	22.00	23.95	31.00	43.00	43.00	26.47
	2.1-7.1	2.2-7.2	2.3-7.3	2.4-7.4	2.5-7.5	2.6-7.6	2.7-7.7	2.8-7.8
Mean ± SD	-0.21 ± 8.20	-2.09 ± 7.39	-3.73 ± 8.25	-7.38 ± 10.38	-9.40 ± 10.95	-3.65 ± 11.34	9.89 ± 14.70	9.54 ± 19.28
p value	0.658	**<0.001**	**<0.001**	**<0.001**	**<0.001**	**<0.001**	**<0.001**	**<0.001**
p2.5	-17.00	-17.00	-18.95	-27.00	-29.95	-22.00	-17.48	-31.47
p97.5	18.47	12.00	12.00	10.47	9.47	26.95	41.47	45.47
	3.1-6.1	3.2-6.2	3.3-6.3	3.4-6.4	3.5-6.5	3.6-6.6	3.7-6.7	3.8-6.8
Mean ± SD	0.45 ± 7.13	-0.14 ± 7.71	1.29 ± 8.92	-1.08 ± 8.35	-4.39 ± 8.34	-2.51 ± 8.82	-1.20 ± 8.52	9.55 ± 15.00
p value	0.275	0.759	**0.013**	**0.025**	**<0.001**	**<0.001**	**0.015**	**<0.001**
p2.5	-14.00	-15.00	-14.47	-18.48	-25.00	-21.00	-18.00	-19.95
p97.5	15.00	15.00	20.00	14.00	11.47	15.00	15.00	40.00
	4.1-5.1	4.2-5.2	4.3-5.3	4.4-5.4	4.5-5.5	4.6-5.6	4.7-5.7	4.8-5.8
Mean ± SD	2.61 ± 5.57	3.41 ± 5.23	5.27 ± 4.72	5.56 ± 12.36	-1.28 ± 11.19	-0.63 ± 4.54	-1.32 ± 4.67	0.88 ± 9.52
p value	**<0.001**	**<0.001**	**<0.001**	**<0.001**	**0.048**	**0.017**	**<0.001**	0.109
p2.5	-8.00	-7.47	-4.47	-17.95	-21.47	-9.00	-10.00	-17.42
p97.5	14.00	13.00	14.00	30.00	20.47	8.00	8.00	24.47

Table S9. Mean ± SD of the thickness difference between corresponding cells of the inferior and superior hemispheres of the outer retina (OUTER) and its statistical significance (p value) (Student's test for paired samples). Positive values indicate that thicker thicknesses are detected in the inferior cells than in its corresponding cells in the superior hemisphere. Negative values indicate that inferior cell show thinner thicknesses than its corresponding cells in the superior hemisphere. 2.5th and 97.5th percentiles of the asymmetry thickness in each cell of the 8x8 grid for the OUTER are also shown.

OUTER	1.1-8.1	1.2-8.2	1.3-8.3	1.4-8.4	1.5-8.5	1.6-8.6	1.7-8.7	1.8-8.8
Mean ± SD	-2.22 ± 3.37	-2.57 ± 2.90	-2.57 ± 2.53	-2.65 ± 2.27	-3.03 ± 2.45	-3.17 ± 2.46	-3.18 ± 2.57	-3.03 ± 3.06
p value	**<0.001**	**<0.001**	**<0.001**	**<0.001**	**<0.001**	**<0.001**	**<0.001**	**<0.001**
p2.5	-7.00	-7.47	-7.47	-7.00	-7.00	-7.47	-8.00	-9.47
p97.5	2.00	1.00	1.00	1.00	1.00	1.00	2.00	4.95
	2.1-7.1	2.2-7.2	2.3-7.3	2.4-7.4	2.5-7.5	2.6-7.6	2.7-7.7	2.8-7.8
Mean ± SD	-1.57 ± 1.90	-1.64 ± 2.00	-1.78 ± 1.80	-1.99 ± 1.83	-2.36 ± 2.02	-2.22 ± 2.31	-2.37 ± 2.09	-2.43 ± 2.93
p value	**<0.001**	**<0.001**	**<0.001**	**<0.001**	**<0.001**	**<0.001**	**<0.001**	**<0.001**
p2.5	-6.00	-5.47	-6.00	-6.00	-6.00	-6.00	-6.47	-8.00
p97.5	2.00	1.47	1.00	1.00	1.00	1.00	2.00	1.47
	3.1-6.1	3.2-6.2	3.3-6.3	3.4-6.4	3.5-6.5	3.6-6.6	3.7-6.7	3.8-6.8
Mean ± SD	-0.95 ± 1.76	-0.93 ± 1.76	-1.05 ± 1.58	-1.28 ± 1.63	-1.75 ± 2.07	-1.52 ± 2.29	-1.24 ± 2.07	-1.22 ± 3.53
p value	**<0.001**	**<0.001**	**<0.001**	**<0.001**	**<0.001**	**<0.001**	**<0.001**	**<0.001**
p2.5	-4.00	-4.00	-4.00	-4.47	-5.47	-5.00	-5.00	-5.47
p97.5	2.00	2.00	1.47	2.00	2.00	2.00	2.00	3.00
	4.1-5.1	4.2-5.2	4.3-5.3	4.4-5.4	4.5-5.5	4.6-5.6	4.7-5.7	4.8-5.8
Mean ± SD	-0.42 ± 1.44	-0.64 ± 1.32	-0.81 ± 1.79	-0.40 ± 2.42	-0.13 ± 2.04	-0.25 ± 1.49	-0.17 ± 1.41	-0.10 ± 2.09
p value	**<0.001**	**<0.001**	**<0.001**	**0.005**	0.910	**0.004**	**0.037**	0.393
p2.5	-3.00	-3.00	-4.00	-4.47	-4.47	-3.00	-3.00	-4.00
p97.5	2.00	1.47	2.00	3.00	4.00	3.00	2.00	3.00

Table S10. Mean ± SD of the thickness difference between corresponding cells of the inferior and superior hemispheres of the complete retina (RETINA) and its statistical significance (p value) (Student's test for paired samples). Positive values indicate that thicker thicknesses are detected in the inferior cells than in its corresponding cells in the superior hemisphere. Negative values indicate that inferior cell show thinner thicknesses than its corresponding cells in the superior hemisphere. 2.5th and 97.5th percentiles of the asymmetry thickness in each cell of the 8x8 grid for the RETINA are also shown.

RETINA	1.1-8.1	1.2-8.2	1.3-8.3	1.4-8.4	1.5-8.5	1.6-8.6	1.7-8.7	1.8-8.8
Mean ± SD	1.48 ± 9.26	0.72 ± 9.57	-1.19 ± 9.80	-2.86 ± 11.09	-1.15 ± 13.37	8.43 ± 14.94	4.53 ± 18.04	-19.52 ± 20.19
p value	**0.006**	0.194	**0.036**	**<0.001**	0.135	**<0.001**	**<0.001**	**<0.001**
p2.5	-17.95	-19.48	-20.00	-23.42	-26.42	-20.00	-32.95	-55.95
p97.5	18.00	18.47	18.47	20.00	25.90	39.00	38.47	24.47
	2.1-7.1	2.2-7.2	2.3-7.3	2.4-7.4	2.5-7.5	2.6-7.6	2.7-7.7	2.8-7.8
Mean ± SD	-1.77 ± 8.26	-3.69 ± 7.65	-5.51 ± 8.51	-9.47 ± 10.64	-11.70 ± 11.08	-5.91 ± 11.50	7.57 ± 14.68	7.17 ± 19.17
p value	**<0.001**	**<0.001**	**<0.001**	**<0.001**	**<0.001**	**<0.001**	**<0.001**	**<0.001**
p2.5	-18.95	-18.48	-20.48	-29.47	-31.00	-24.00	-19.48	-33.95
p97.5	14.47	10.47	10.47	9.47	7.47	24.00	39.00	43.47
	3.1-6.1	3.2-6.2	3.3-6.3	3.4-6.4	3.5-6.5	3.6-6.6	3.7-6.7	3.8-6.8
Mean ± SD	-0.46 ±7.31	-1.15 ± 7.86	0.24 ± 9.06	-2.33 ± 8.53	-6.15 ± 8.77	-4.04 ± 9.25	-2.44 ± 8.78	8.35 ± 14.91
p value	0.276	**0.012**	0.647	**<0.001**	**<0.001**	**<0.001**	**<0.001**	**<0.001**
p2.5	-16.00	-16.00	-16.48	-20.00	-26.00	-23.00	-20.00	-20.00
p97.5	14.00	14.00	17.47	13.95	9.95	13.95	15.00	39.47
	4.1-5.1	4.2-5.2	4.3-5.3	4.4-5.4	4.5-5.5	4.6-5.6	4.7-5.7	4.8-5.8
Mean ± SD	2.21 ± 5.72	2.79 ± 5.51	4.41 ± 4.81	5.07 ± 11.61	-1.33 ± 10.40	-0.79 ± 4.61	-1.46 ± 4.65	0.79 ± 9.65
p value	**<0.001**	**<0.001**	**<0.001**	**<0.001**	**0.027**	**0.003**	**<0.001**	0.155
p2.5	-7.00	-8.00	-5.47	-18.00	-20.95	-9.00	-10.47	-15.47
p97.5	13.00	12.00	13.47	28.47	19.00	9.00	7.47	26.00
